# Supplementary material for: Causal interplay between lactose intolerance and gut microbiota: a combined bidirectional Mendelian randomization and in vivo validation study
Source: Front Nutr. 2026 Jun 1;13:1803337. doi: 10.3389/fnut.2026.1803337 (PMC13265576; doi:10.3389/fnut.2026.1803337)
Supplement: Supplementary file 1 [file Data_Sheet_1.zip › supplementary materials/Forward/forest plot/ebi-a-GCST90027664.finngen_R12_E4_LACTONAS.pdf]

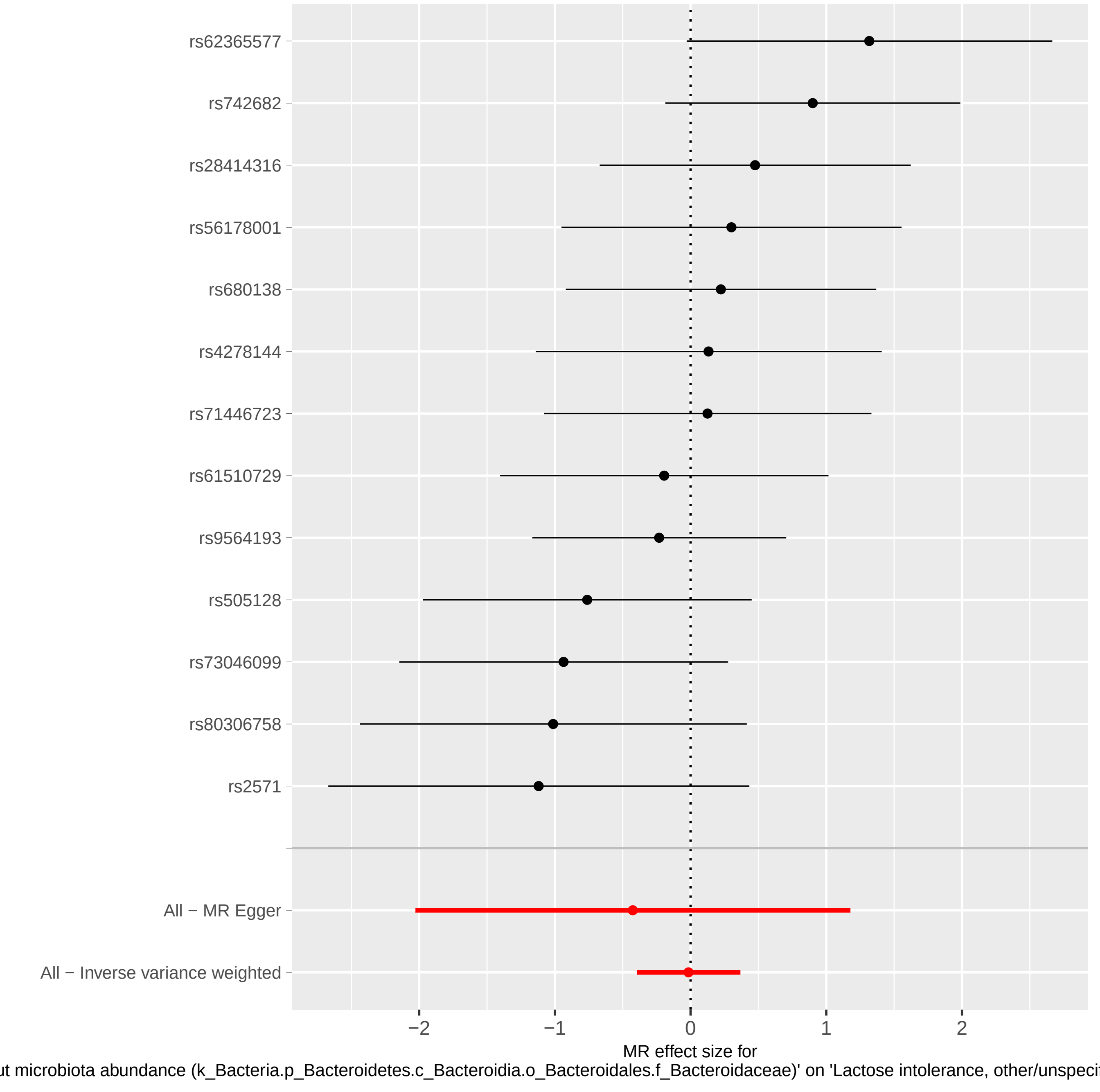
rs62365577
rs742682
rs28414316
rs56178001
rs680138
rs4278144
rs71446723
rs61510729
rs9564193
rs505128
rs73046099
rs80306758
rs2571
All − MR Egger
All − Inverse variance weighted
−2
−1
0
1
2
MR effect size for
ut microbiota abundance (k_Bacteria.p_Bacteroidetes.c_Bacteroidia.o_Bacteroidales.f_Bacteroidaceae)' on 'Lactose intolerance, other/unspeci
